# Molecular monitoring of short- and long-term transcriptional effects of hair growth stimulating agents

**DOI:** 10.1371/journal.pone.0316128

**Published:** 2024-12-23

**Authors:** Sabrina K. Henne, Lara M. Hochfeld, Werner Bartmann, Thomas Welss, Markus M. Nöthen, Stefanie Heilmann-Heimbach

**Affiliations:** 1 Institute of Human Genetics, School of Medicine & University Hospital Bonn, University of Bonn, Bonn, Germany; 2 Henkel Consumer Brands, Düsseldorf, Germany; Concordia University Irvine, UNITED STATES OF AMERICA

## Abstract

Male-pattern hair loss (MPHL) is the most common form of hair loss in humans. Limited treatment options exist, which are not curative and vary in efficacy and invasiveness. Therapeutic and cosmetic hair growth stimulating agents that alleviate hair loss at a low risk of side effects are therefore of interest. The efficacy of hair growth-stimulating agents is mainly evaluated by hair comb tests and trichograms. These methods do not offer molecular insights, which can provide early insights into treatment response and may be useful in monitoring long-term compliance and efficacy. We propose a general concept for the molecular monitoring of hair growth stimulating agent treatment response in vivo, based on RNA and microRNA expression profiling before and during treatment. The molecular profile can be extended by individual genotype information to assess the impact of genetic constitution on treatment response. To test this methodological approach, 91 male participants with visible signs of and/or a family history of MPHL were assigned to four groups to investigate the effects of three hair growth stimulating agents versus placebo. mRNA- and microRNA-Seq was performed on plucked hair follicle samples before, after four days, and after six weeks of treatment. Genotyping was performed on DNA extracted from blood or saliva samples. Differential expression analyses identified 52 differentially expressed genes and 17 modulated pathways following treatment with the three hair growth stimulating agents. While the majority of effects were detectable after 6-week treatment, 23% of genes showed significant regulation after 4-day treatment. Integration with genetic data through pathway-based polygenic risk score analyses identified 5 associations between genetic background and treatment effects, pointing to a potential value of companion diagnostics for hair growth stimulating agents. Our data show that this molecular monitoring approach provides insights into hair growth stimulating agent treatment response as early as days within commencing treatment, and is suitable to monitor long-term treatment effects and compliance. Combined with genetic profiling, this approach may enable personalized prediction of treatment efficacy and compliance.

## Introduction

Male-pattern hair loss (MPHL) is a highly heritable and progressive form of hair loss with a lifetime prevalence of ~80% in European men [1]. Affected men can experience psychological consequences, especially when affected from a young age [2]. While significant progress has been made in the identification of the underlying genetic factors and biological pathways that contribute to MPHL, few treatment options for the androgen-dependent hair loss exist.

The only FDA-approved drugs are oral finasteride and topical minoxidil. These drugs vary in their efficacy, require early onset of treatment, are not curative and can induce severe side effects, including potentially persistent sexual dysfunction and depressive mood changes after treatment with oral finasteride or contact dermatitis and facial hypertrichosis after treatment with topical minoxidil [3, 4]. Surgical transplantation of hairs from the non-balding occipital scalp to balding areas of the scalp as well as scalp injections with platelet-rich plasma exists as an alternative treatment, although this procedure is invasive and requires a subsequent recovery time [3, 5, 6]. The 2020 global market size of androgenetic alopecia treatments has been estimated at USD 1.6 billion [7] and a survey among alopecia patients found the willingness to undergo treatment to be highest for topical (74%) and oral (64%) treatment options [8]. These data highlight the potential value of efficacious and well-tolerated MPHL therapeutics or cosmetic hair growth products. Moreover, early treatment of MPHL with such treatment options could delay hair loss sufficiently long to obviate the desire for more invasive therapies.

A critical step in the development of hair growth agents is the evaluation of treatment efficacy and safety of formulations. To date, various in vivo and ex vivo methods for treatment evaluation exist. Non-invasive methods such as visual inspection, hair comb test and hair pull test are used to evaluate hair thinning and hair fall severity. The trichogram, a minimally invasive technique involving microscopy of plucked small hair bushels, is used to evaluate hair density and anagen-to-telogen ratio [9, 10]. However, these methods do not provide molecular-level insights, which could offer early indications of treatment response and may be useful in monitoring efficacy and side effects of long-term treatment. Ex-vivo hair follicle cultures can be used in this regard, though they may be limiting in the research questions they can answer, as the hair follicles are removed from their natural neural, vascular and endocrine environment that influences hair follicle biology. This is, for instance, a possible reason why the hair growth-stimulating effects of minoxidil observed in vivo could not be replicated in hair follicle cultures [11].

Previous studies have employed transcriptome profiling, including mRNA- and microRNA (miRNA)-sequencing, to investigate MPHL at a molecular level. These studies, for example, have compared transcriptomic profiles of dermal papillae or plucked hair follicles between affected and unaffected scalp regions. Differentially expressed mRNAs and miRNAs have been linked to androgen signaling, WNT signaling, adipogenesis, and other processes implicated in MPHL pathophysiology [12–14]. These insights highlight the potential of mRNA- and miRNA-Seq for not only advancing our understanding of MPHL etiology, but potentially also for evaluating therapeutic responses. As our knowledge regarding MPHL etiology and the underlying genetic basis increases, insights at the molecular level may be useful in evaluating the efficacy of cosmetic hair growth formulations down to a personalized level. The latter can be achieved by taking into account patients’ genetic backgrounds to predict the extent to which different etiological mechanisms of MPHL are of relevance in certain patients. These patient stratification strategies may then be leveraged to predict efficiency and adverse effects of cosmetic formulations and therapeutics [15]. Here, we propose a concept for the molecular monitoring of hair growth-stimulating agent treatment response in vivo based on mRNA- and miRNA expression profiling of plucked hair follicles. These analyses can be performed alongside trichograms with no added invasiveness to obtain insights at a molecular level. The expansion of the molecular monitoring through genotypic profiling further enables analyses investigating how individual genetic constitution impacts treatment response. To this end, we (i) collected plucked human hair follicle samples before treatment, after short-term treatment (4 days) and after long-term treatment (6 weeks) with hair growth-stimulating agents (hereafter referred to as serums), as well as blood or saliva samples of 89 study participants, (ii) performed mRNA and miRNA sequencing of hair follicle RNA extracts to identify differentially regulated genes and modulated biological pathways, and (iii) performed genotyping analyses using DNA from blood or saliva samples in order to investigate whether genetic factors, represented by polygenic risk scores (PRS) for MPHL influence the serum-mediated effects.

## Materials and methods

### Recruitment and sample collection

Ethical approval was obtained from the ethics committee of the Medical Faculty of the University of Bonn. Study participants were recruited from 4 February 2019 to 15 April 2019. All study participants provided written informed consent. A total of 98 men were recruited into the study. The inclusion criteria were (i) visible signs of MPHL (Hamilton-Norwood scale II-IV) or a familial predisposition for MPHL (i.e., a minimum of two male relatives affected by MPHL), European ancestry as well as no use of hair growth-stimulating agents in the past six months. The clinical characteristics of the study participants included in the analyses are shown in S1 Table.

Each study participant was randomly assigned one of three hair growth-stimulating serums (hereafter named serums A, B or C) or a placebo (i.e. the ethanol-based solvent used in serums A-C). Serum A and B are based on plant extracts and serum C is a commercially available cosmetic hair-loss preventing serum containing a pyrimidine derivative. Hair follicle samples were plucked before treatment, after four days of treatment, and after six weeks of treatment (hereafter referred to as baseline, short-term, and long-term treatment, respectively). Additional details are reported in S1 Appendix. Hair follicle samples were stored in RNAlater stabilization solution (Invitrogen™) at -80°C until nucleic acid extraction. EDTA blood or saliva samples were taken at the baseline visit and stored at -20°C or room temperature, respectively.

### Nucleic acid extraction, sequencing and genotyping

Total RNA was extracted from hair follicle samples using the Qiagen miRNeasy Micro Kit after tissue homogenization. Per treatment group, samples from 21 participants with the best average RNA concentration and purity were selected for sequencing. mRNA and small RNA library preparation were performed using the Lexogen QuantSeq 3’ FWD Library Prep Kit and Small RNA-Seq Library Prep Kit, respectively. Sequencing was performed at 50 bp SE on a HiSeq2500 in High Output mode using v4 reagents. DNA was extracted from EDTA blood samples using the Chemagic Magnetic Separation Module I or from saliva samples collected using the Oragene OG-500 kit followed by extraction according to the Oragene protocol. Samples were genotyped on the Infinium Global Screening Array-24 (+MD) v3.0 BeadChip. Further details are available in S1 Appendix.

### Bioinformatic processing

RNA-Seq data processing and analysis were performed using the Partek Flow analysis software (v9.0) with GRCh38 genome reference data from Ensembl (v99) [16, 17]. Total RNA data processing comprised standard read quality control, alignment to the human genome using the STAR aligner (v2.5.3a), gene-based feature counting using the Partek Expectation-Maximization algorithm, and removal of outliers detected via principal component analysis (PCA). mRNA genes with low expression (counts per million (CPM) < 1.5 in at least 75% of samples) were removed from the analyses and the data were TMM-normalized.

The miRNA data were processed by applying standard read quality control and a two-step read alignment using the BWA backtrack algorithm. The two-step process aimed to remove contaminating ribosomal RNA (rRNA) by first aligning to human rRNAs. Unaligned reads were then aligned to mature miRNAs in the miRbase database (v22.1) [18]. Feature counting, outlier removal, expression filtering (CPM < 1.6 in at least 75% of samples), and TMM-normalization were subsequently applied. Further details on mRNA and miRNA processing are available in S1 Appendix.

### Differential expression analysis

For each of the eight treatment groups (serums A-C and placebo with two treatment durations each), differential expression was calculated using ANOVA. Differential expression was calculated separately for the mRNA and miRNA analyses. The ANOVA was performed by comparing expression after short- or long-term treatment to the baseline of the same individuals. The statistical model accounted for batch effects (extraction and sequencing batch) and individual-treatment interactions. P-values were adjusted for multiple testing using the Benjamini-Hochberg False Discovery Rate procedure (FDR).

### Gene set analysis

Gene set analysis was performed to test for enrichment of regulated mRNA and miRNA genes in biological pathways. mRNA gene set analysis was performed with Enrichr [19] using the WikiPathways 2021 library. The miRNA gene set analysis was performed with miEAA (v2.1) [20] using the miRPathDB WikiPathways category. To obtain a more comprehensive overview of serum-mediated effects on pathways, differentially expressed (DE) genes significant without correction for multiple testing (P ≤ 0.05) were used as input. Any genes that were DE in the placebo group were removed to correct for potential effects of the serum solvent. All expressed mRNA or miRNA genes were supplied as a background reference in the respective analysis.

### PRS analyses

Pathway-based polygenic risk scores (pPRS) were calculated based on participants’ imputed genotype data via PRSice-2 PRSet (v2.3.5) [21, 22] based on single nucleotide polymorphisms (SNPs) showing suggestive association with MPHL (P < 5 × 10^−7^) in a published genome-wide association study (GWAS) [23]. The gene sets used were sourced from the Enrichr [19] WikiPathways 2021 library, and gene boundaries were defined based on the Ensembl GRCh37 gene annotation (v87) [17]. Association testing between pPRS and treatment effects was performed using linear regression via the MatrixEQTL package (v2.3) in R (v4.2.2). Per treatment group, linear regression was performed for each mRNA and miRNA gene, correlating the expression difference between treatment and baseline (as log_2_ fold change) with one pPRS at a time. Only genes showing differential expression (P ≤ 0.05) upon treatment with the corresponding serum (either short- or long-term treatment) were tested for each treatment group. P-values were adjusted for multiple testing using FDR.

## Results and discussion

Of the 98 men enrolled in the study, 91 attended at least the first sample collection visit. 89 men attended all three sample collection visits, resulting in an overall study compliance of 91%. For the molecular analyses, a total of 84 sample sets were selected for sequencing based on nucleic acid quantity and purity. Each sample set corresponded to three samples of different time points, with 21 sample sets being chosen per treatment group. Of these, 81 and 78 sample sets passed sequencing and post-processing quality control in the mRNA and miRNA analysis, respectively. As a result, the data sets for the different treatment groups comprised data from 17 to 21 individuals (S2 Table).

**Fig 1:**
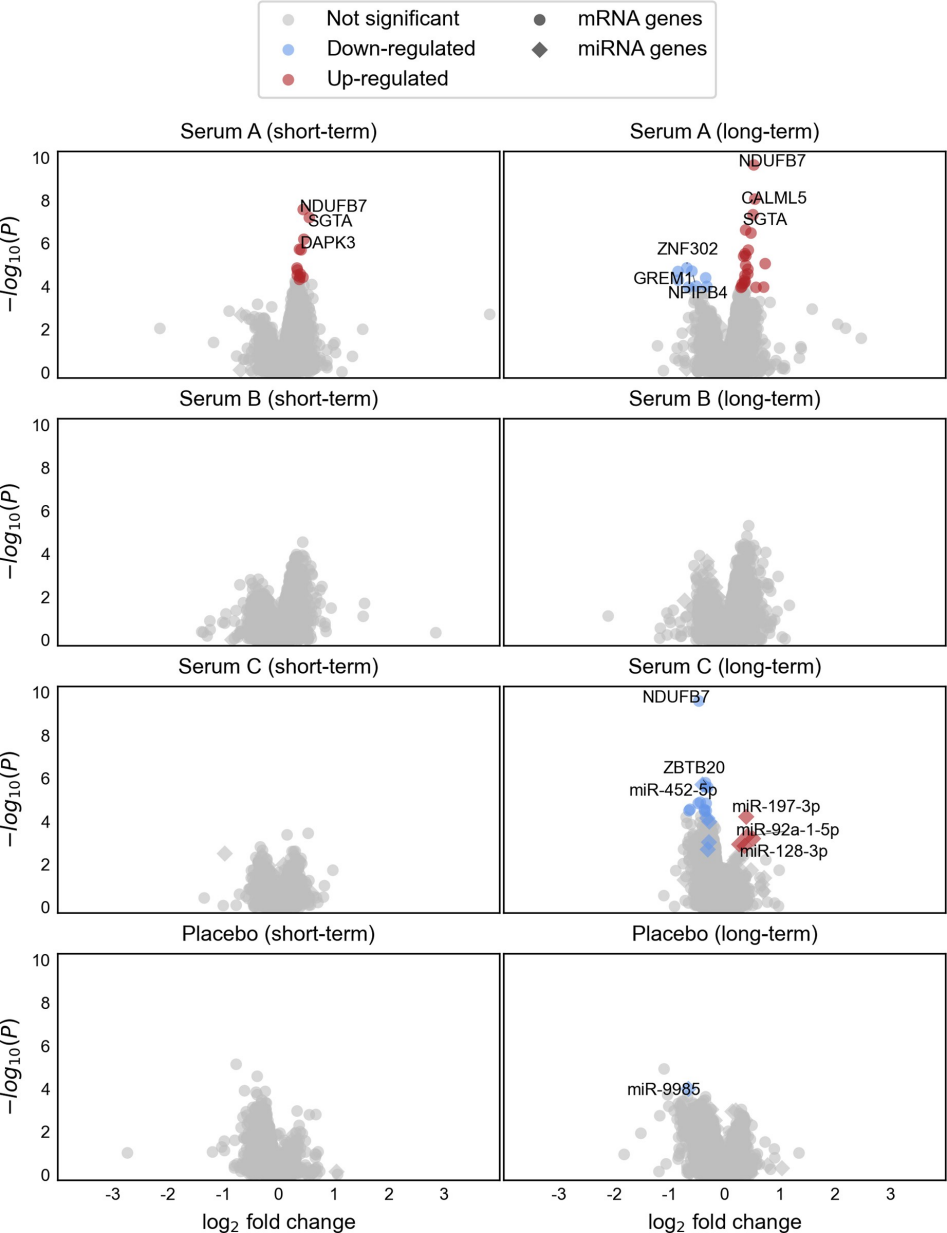
Combined results of the differential mRNA and miRNA expression analyses for the treatment groups. Dots represent hair follicle-expressed mRNA genes and diamonds represent hair follicle-expressed miRNA genes. Differentially expressed genes (FDR ≤ 0.05) are depicted in blue (downregulated upon treatment) or red (upregulated upon treatment). The top three differentially expressed genes (up- and downregulated) per treatment group are annotated.

**Fig 2:**
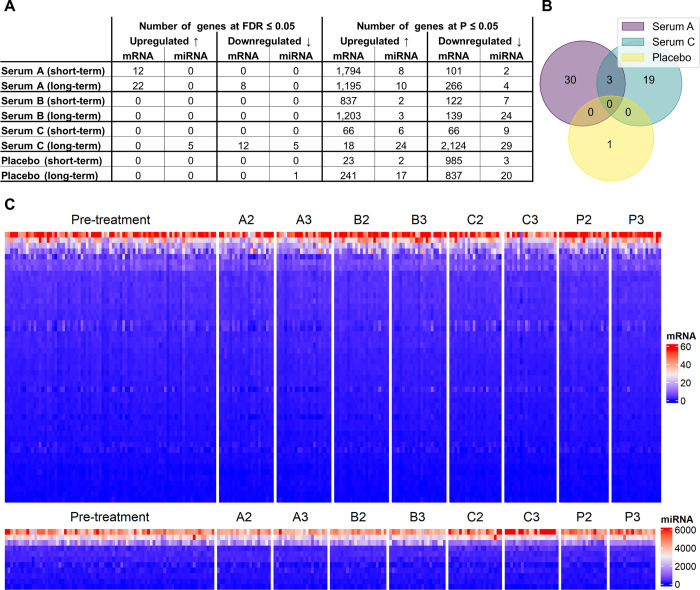
Overview of differential expression results. (A) Numbers of differentially expressed mRNA and miRNA genes (FDR ≤ 0.05 or P ≤ 0.05) across treatment groups. (B) Venn diagram of differentially expressed genes overlapping between serum A, serum C and placebo (both short- and long-term treatment). (C) Heat maps representing normalized expression of mRNA (above) and miRNA (below) genes across treatment groups. Treatment groups refer to serums A, B, C and placebo (P), at time points 2 (short-term treatment) and 3 (long-term treatment). Only genes differentially expressed in at least one treatment group are shown.

Using the molecular monitoring approach on human hair follicles after treatment with three hair growth serums or placebo, we detected differential expression of a total of 53 mRNA and miRNA genes (FDR ≤ 0.05, Figs 1 and 2, S3 Table). Serum B showed no significant molecular effects. In the placebo group, only the gene *hsa-miR-9985* was detected to be downregulated upon long-term treatment, with the serum groups showing downregulation of the same gene at a nominally significant level (P ≤ 0.05, FDR > 0.05) after long-term treatment.

Several of the differentially expressed genes have been previously implicated in hair (loss) biology, supporting the applicability of molecular monitoring in this context. For instance, *NDUFB7* is involved in electron transport chain activity, which has been shown to be reduced in balding human dermal papilla cells (DPCs). *NDUFB7* was further found to be upregulated in DPCs after stimulation with dihydrotestosterone [24]. In our analyses, it was the most strongly differentially expressed gene in serum A both after short-term (FDR = 3.2×10^−4^, fold change [FC] = -1.4) and long-term treatment (FDR = 2.7×10^−6^, FC = -1.4) and in serum C after long-term treatment (FDR = 3.1×10^−6^, FC = 1.4), indicating a potential influence on mitochondrial function. *GPX4*, which was upregulated upon long-term treatment with serum C (FDR = 8.0×10^−3^, FC = 1.2), has been shown to result in dysmorphic hair follicles and persisting sparse hair when disrupted in mice, indicating an important role in hair morphogenesis and hair follicle cycling [25].

Among miRNAs, miR-221-3p was upregulated in response to long-term treatment with serum C (FDR = 5.7×10^−3^, FC = 1.2). miR-221 was previously found to be upregulated in balding DPCs [26] and has been implicated as a target of androgen receptor signaling, suppressing IGF-1 [27]. It is unclear whether this could indicate an unintended effect that potentially counteracts the desired outcome of stimulating hair growth.

Notably, we also observed regulation of *DAPK3* (Serums A and C) and *ZBTB20* (Serum C), which have been identified as potential candidate genes for MPHL based on GWAS [23, 28]. Several differentially expressed genes have further been implicated in other forms of hair loss, including alopecia areata (*CALML5* [29], *GNAS* [30]), senescent alopecia (*CALML5* [31], *ITGA2* [31]), lichen planopilaris (*MVD* [32]), female-pattern hair loss (miR-197-3p and miR-92a-1-5p [33]), and chemotherapy-induced hair loss (*CDK2* [34]).

Regarding treatment duration, the majority of differentially expressed genes were detected after long-term treatment of six weeks (50 genes FDR ≤ 0.05). Early regulation (after 4-day treatment) was observed in 12 genes in serum A, with effects on nine of these genes persisting until six weeks after commencing treatment. A total of 21 genes were regulated only upon long-term treatment with serum A, but differential expression of 18 of these genes was already present at a nominally significant level (P ≤ 0.05, FDR > 0.05) after short-term treatment. Serum C distinctly showed effects only after long-term treatment (22 genes FDR ≤ 0.05), with differential expression of three of these genes being present at a nominally significant level (P ≤ 0.05, FDR > 0.05) after short-term treatment. Together, these findings show that molecular monitoring can detect molecular effects of hair growth serums as early as four days within commencing treatment, before trichoscopic changes are to be expected. The inclusion of different time points may be useful to pinpoint optimal treatment durations. For instance, in serum C, molecular effects were detected only after long-term treatment. As this serum’s recommended application duration is 6 weeks, molecular monitoring confirms that this treatment duration is necessary. Additionally, further long-term follow-up could help to detect signs of potentially decreasing effectiveness of hair growth-stimulating serums and to identify possible molecular causes.

**Fig 3:**
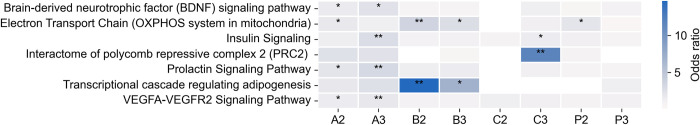
mRNA gene set enrichment results of selected pathways with previous evidence for a role in hair biology or MPHL. Only pathways which are significantly enriched (FDR ≤ 0.05) in at least one treatment group are shown. Treatment groups refer to serums A, B, C and placebo (P), at time points 2 (short-term treatment) and 3 (long-term treatment). ** indicates significant enrichment (FDR ≤ 0.05), * indicates nominally significant enrichment (P ≤ 0.05). The enrichment odds ratio is shown on a color scale.

Gene set enrichment analyses of a less stringent set of differentially expressed mRNA and miRNA genes (P ≤ 0.05) detected a total of 17 modulated pathways (FDR ≤ 0.05) across the three serums (S3 and S4 Figs). Effects on six pathways were detected in the placebo group, including one pathway modulated in the serum groups. It is therefore recommendable to include a suitable placebo in a molecular monitoring study. The pathways modulated in the serum groups included several pathways previously implicated in MPHL (Fig 3). A notable example is prolactin (PRL) signaling, which was found to be regulated in serum A. PRL signaling is a known regulator of the hair cycle, and has been implicated in MPHL through a risk locus located nearby the *PRL* gene [35]. Similarly, adipogenesis, which was regulated in serum B, was identified via GWAS on MPHL [36], and adipogenic markers have been found to be downregulated in adipose tissue of the balding scalp [37]. Brain-derived neurotrophic factor (BDNF) signaling, enriched among miRNA target genes in serum C, has been shown to be upregulated in balding DPCs and to negatively impact hair growth and hair follicle cycling in mice [38]. Further modulated pathways and relevant mechanisms include VEGFA / VEGFR2 signaling (serum A, implicated in vascularization [39]), polycomb repressive complex 2 function (serum C, implicated in hair cycling and growth [40]), the electron transport chain function (serum B, suggested to be deficient in MPHL [24]), WNT signaling (serum C, implicated in hair cycling and in MPHL etiology [41]), and insulin- or insulin-like growth factor signaling (serums A and C, implicated in hair cycling and MPHL [35]). Together, these findings suggest that the molecular monitoring approach can identify specific and biologically plausible pathways through which the serums may exert their effects, providing mechanistic insights into their actions.

Insights gained from additional genetic analyses may pave the way for personalized cosmetics, in which hair growth-stimulating serums could be chosen to address patients’ treatment needs based on their genetic background. Differential expression data were integrated with genetic data using pPRS analyses to identify associations between the study participants’ genetic background at the pathway-level and serum-mediated effects on gene expression. The analyses detected 5 associations (FDR ≤ 0.05, S4 Table) between pPRS and serum-mediated expression differences in DE mRNA or miRNA genes (P ≤ 0.05 in any of serum A, B or C). For example, upon short-term treatment with serum A, MPHL-associated genetic variants in signaling pathways of sphingosine-1-phosphate G protein-coupled receptors enhance upregulation of *WNT5A*, which has previously been shown to inhibit telogen-to-anagen transition of hair follicles [42]. While we extracted DNA for genotyping from blood or saliva samples, DNA could also be extracted from hair follicles, obviating the need for additional sample types.

In conclusion, using a minimally invasive method for RNA-Seq of plucked hair follicles, we were able to identify mRNA genes, miRNA genes and pathways modulated by three tested cosmetic hair growth stimulating agents, as well as interplaying genetic effects through pPRS. While this was an explorative study with a limited sample size, we believe that such molecular monitoring analyses can be useful in identifying molecular effects of cosmetic hair growth agents, and can for instance be performed alongside trichograms to expand and accelerate insights into treatment response with no additional invasiveness. This study achieved a relatively high compliance of 91%, which was likely due to a limited study duration and the absence of severe side effects. As molecular monitoring can detect early effects, their integration into study designs can be beneficial in terms of time- and cost-efficiency and may improve study compliance. Molecular insights can further help to understand and develop additional formulations and, in combination with genetic analyses, may eventually enable personalized prediction of treatment efficacy. While we applied this method to MPHL, it is transferrable to other forms of hair loss, such as female-pattern hair loss, as well.

## Supporting information

S1 Appendix Supplementary methods. (DOCX)

S1 Table Clinical characteristics of study participants. Age, Hamilton-Norwood grade and assigned serum of each study participant in the final sample set used in the analyses.
(DOCX)

S2 Table Number of final sample sets. Number of complete sample sets (corresponding to samples of the same study participant at three different time points) passing sequencing and post-processing quality control per serum and analysis (mRNA/microRNA).
(DOCX)

S3 Table Overview of significantly differentially expressed genes. Significantly differentially expressed mRNA or miRNA genes (FDR ≤ 0.05) identified in the differential expression analyses and their FDR- and fold change-values across treatment groups. The treatment groups serum B short-term, serum B long-term, serum C short-term and placebo short-term are not shown, as these showed no differentially expressed genes. Missing values indicate the gene did not meet the expression threshold in that specific treatment group and was therefore not included in the analysis. FC–Fold change.
(DOCX)

S4 Table Significant associations of the pathway-based polygenic risk score analyses. Significant associations (FDR ≤ 0.05) identified between pathway-based polygenic risk scores (pPRS) and serum-mediated effects on gene expression of DE mRNA or miRNA genes (P ≤ 0.05 in any of serum A, B or C).
(DOCX)

S1 Fig Schematic overview of the sampling process. (A) Hair follicles were extracted from participants at three visits. On the first visit, a blood sample was drawn, personal information was recorded and participants were given a serum or placebo to be used for the following six weeks. On the third visit, participants answered a questionnaire on their experience with the serum. (B) Schematic image of the location from which hair follicles were sampled, as denoted by the blue arrow. Approximately 50 hair follicles were plucked from the center of the scalp.
(TIFF)

S2 Fig Schematic overview of mRNA and microRNA sequencing data preprocessing steps. (TIFF)

S3 Fig Full gene set enrichment results based on mRNA genes. Only pathways which are significantly enriched (FDR ≤ 0.05) in at least one treatment group are shown. Treatment groups refer to serums A, B, C and placebo (P), at time points 2 (short-term treatment) and 3 (long-term treatment). ** indicates significant enrichment (FDR ≤ 0.05), * indicates nominally significant enrichment (p ≤ 0.05). The enrichment odds ratio is shown on a color scale.
(TIFF)

S4 Fig Full gene set enrichment results based on microRNA genes. Only pathways which are significantly enriched (FDR ≤ 0.05) in at least one treatment group are shown. Treatment groups refer to serums A, B, C and placebo (P), at time points 2 (short-term treatment) and 3 (long-term treatment). ** indicates significant enrichment (FDR ≤ 0.05), * indicates nominally significant enrichment (p ≤ 0.05). The enrichment odds ratio is shown on a color scale.
(TIFF)
