# Supplementary material for: Molecular monitoring of short- and long-term transcriptional effects of hair growth stimulating agents
Source: PLoS One. 2024 Dec 23;19(12):e0316128. doi: 10.1371/journal.pone.0316128 (PMC11666053; doi:10.1371/journal.pone.0316128)
Supplement: S1 Appendix — (DOCX) [file pone.0316128.s001.docx]

# **S1 Appendix: Supplementary Methods**

## Sample and data collection

An overview of the study design in regards to sample and data collection is shown in Supplementary Figure 1. Hair follicle samples were taken at three visits. At each visit, hair follicles were plucked with sterile forceps and kept in 700 μL RNAlater stabilization solution (Invitrogen™) at -20°C until further processing. Each sample comprised approximately 50 hairs plucked with sterile forceps from the frontal-occipital transition area at the center of the scalp, as participants were likely to apply a sufficient amount of hair serum to this area.

At their first appointment, participants were given 150 mL of a randomly assigned serum or the placebo, as well as a corresponding application note. Participants were instructed to use the serum at the same time each day for 6 weeks. 3 mL of serum was to be evenly applied and massaged onto the scalp for 30 seconds. Participants were further instructed not to wash their hair for 6 hours after application and not to use other hair growth-stimulating or dyeing products. Participants’ personal information was recorded and a blood or saliva sample was taken for genotyping at the first visit. At their final appointment after six weeks of treatment, participants were additionally asked to answer a questionnaire concerning their personal experience with the serum.

## RNA extraction

Extraction of total RNA (mRNA and microRNA) was performed using the miRNeasy Micro kit. In order to minimize batch effects, the three samples of a participant were always processed within one batch. As samples required fast processing to prevent RNA degradation, a maximum of twelve samples were processed at once. Crystals arising from the RNA*later* solution were removed and the plucked hairs were placed in a tube containing 700 μL QIAzol and six 2.8mm ceramic beads. The hair follicles were lysed using a Precellys tissue homogenizer for 2 x 10 seconds at 5000 revolutions per minute. Further steps of the RNA extraction were performed as described in the miRNeasy Micro kit manufacturer’s protocol without DNase-digestion. RNA concentration and purity of samples was measured on a NanoDrop device using 1 μL sample volume. The samples were stored at -80°C.

## mRNA and microRNA sequencing

Per serum, 21 participants whose samples had the best average RNA concentrations and purity (based on the 260/230 ratio) were chosen for sequencing (n = 252 samples). Total RNA samples were thawed on ice and diluted to a concentration of 30 ng/µl with RNAse-free water based on the concentration measured by NanoDrop. Library preparation and sequencing were performed by the Next Generation Sequencing Core Facility of the University Hospital of Bonn. For the mRNA sequencing, 50 ng per sample were used as starting material for the library preparation and indexing using the QuantSeq 3’ FWD Library Prep Kit according to the manufacturer’s protocol. For the microRNA sequencing, 65 ng per sample were used as starting material for the library preparation and indexing using the Small RNA-Seq Library Prep Kit according to the manufacturer’s protocol. Quality control before library preparation, before pooling and after pooling was carried out with the TapeStation4200 system using the D1000 Screen Tape assay. Input volumes for pooling were based on TapeStation concentration measurements and a target molarity of 2 nM. For microRNA sequencing, nucleic acid molecule sizes were determined from the TapeStation measurements and pooling volume was based on the concentration of nucleic acids sized 135 bp to 170 bp. 50 bp single-end sequencing was performed on a HiSeq 2500 in High Output mode using v4 reagents.

## DNA extraction and genotyping

DNA was automatically extracted from participants’ EDTA whole blood samples using the Chemagic Magnetic Separation Module I according to the manufacturer’s protocol. Purified DNA was stored at -80°C. 200 ng purified genomic DNA was used as input for genotyping, which was performed on the Infinium Global Screening Array-24 (+MD) v3.0 BeadChip using the Infinium HD Assay Kits and the BeadArray platform according to the manufacturer’s protocol. The resulting measurements were analyzed using the GenomeStudio software.

## Data preprocessing

Demultiplexing of sequencing data as well as trimming of Illumina adapters was performed using the tool bcl2fastq. Further processing and analysis were performed within the Partek Flow analysis software using genomes and annotation files by Ensembl (release 99). A schematic overview of the mRNA and microRNA preprocessing steps are shown in Supplementary Figure 2.

### mRNA data preprocessing

Low-quality bases (Q <10) were trimmed from the 3’ end of reads and reads < 20 bp were removed. The STAR aligner (v2.5.3a) was utilized to map reads to the human genome, using the Ensembl primary assembly (GRCh38.p13) as a reference genome. The Ensembl feature annotation was used in the alignment as a source of information on splice sites. The following non-default parameters for STAR were set:

−−outFilterType BySJout
−−outFilterMultimapNmax 20
−−outFilterMismatchNmax 999
−−alignSJoverhangMin 8
−−alignSJDBoverhangMin 1
−−outFilterMismatchNoverLmax 0.1
−−alignIntronMin 20
−−alignIntronMax 1000000

The mapped reads were assigned to genes and counted using the Partek Expectation-Maximization algorithm and the Ensembl feature annotation with the following parameters:

Require junction reads to match introns: True

Strand specificity: Forward

Minimum read overlap with feature: 80%

Min reads across all samples: 10

### microRNA data preprocessing

First, the Lexogen adapter was trimmed from the 3’ microRNA read end. Low quality bases (Q < 20) were trimmed from both read ends and reads < 15 bp were removed. The BWA backtrack algorithm was employed for read alignment in a two-step process aiming to remove contaminating rRNA. First, reads were aligned to human ribosomal RNA (rRNA), using a subset of entries from the Ensembl feature annotation as a reference. Remaining unmapped reads were subsequently aligned to mature microRNAs in the miRBase v22.1 database. The following non-default parameters were set:

-n 1
-o 0

-e 0

The mapped reads were assigned to mature microRNAs and counted using the Partek Expectation-Maximization algorithm and mature microRNAs in the miRBase feature annotation. The following non-default parameters were set:

Require junction reads to match introns: False

Strand specificity: Forward

Minimum read overlap with feature: 100%

Min reads across all samples: 5

Principal component analyses (PCA) were performed in order to detect and remove outliers from further analyses. In the case of microRNA, outliers were additionally removed based on low numbers of reads (< 200,000). Outliers were removed at the participant-level (i.e. all samples stemming from the same participant as the outlier sample).

### Genotyping data preprocessing

Quality-controlled genotypes were exported from Genome Studio to PLINK format using the TOP strand as strand specification. Strands were flipped according to the GSAMD-24v3-0 manifest to obtain the forward strand orientation.

Variants were filtered in PLINK (v1.9) for a minor allele frequency of 0.01, maximum missing call rates of 0.1 and Hardy-Weinberg equilibrium exact test p-values > 10^-6^. Duplicate variants were removed, Illumina SNP IDs were changed to rsIDs using the Infinium Global Screening Array Loci to rsID conversion file and reference/alternative allele information was adapted to the dbSNP annotation. The resulting VCF file was pre-phased and imputed with EAGLE2+PBWT using the Haplotype Reference Consortium reference panel (release 1.1).
