# Supplementary material for: Molecular monitoring of short- and long-term transcriptional effects of hair growth stimulating agents
Source: PLoS One. 2024 Dec 23;19(12):e0316128. doi: 10.1371/journal.pone.0316128 (PMC11666053; doi:10.1371/journal.pone.0316128)
Supplement: S1 Table — Age, Hamilton-Norwood grade and assigned serum of each study participant in the final sample set used in the analyses. (DOCX) [file pone.0316128.s002.docx]

| **Participant ID** | **Age** | **Hamilton-Norwood grade** | **Assigned serum** |
| --- | --- | --- | --- |
| 1 | 35 | III | A |
| 3 | 35 | IV | A |
| 7 | 20 | II | A |
| 10 | 29 | II | A |
| 14 | 25 | II | A |
| 18 | 37 | III vertex | A |
| 25 | 32 | II | A |
| 29 | 32 | III | A |
| 36 | 33 | II | A |
| 40 | 25 | II | A |
| 44 | 41 | III vertex | A |
| 47 | 24 | II | A |
| 51 | 33 | III vertex | A |
| 55 | 37 | IVA | A |
| 59 | 23 | IIA | A |
| 63 | 38 | IV | A |
| 67 | 29 | III vertex | A |
| 69 | 46 | III vertex | A |
| 73 | 27 | II | A |
| 77 | 23 | III vertex | A |
| 80 | 26 | IVA | A |
| 4 | 24 | II | B |
| 8 | 32 | IVA | B |
| 11 | 22 | II | B |
| 15 | 28 | II | B |
| 19 | 25 | II | B |
| 22 | 39 | III vertex | B |
| 26 | 34 | III vertex | B |
| 30 | 23 | II | B |
| 33 | 32 | III vertex | B |
| 37 | 34 | II | B |
| 41 | 27 | III vertex | B |
| 45 | 37 | III vertex | B |
| 48 | 28 | III vertex | B |
| 52 | 32 | III vertex | B |
| 56 | 27 | IV | B |
| 60 | 33 | IIA | B |
| 64 | 31 | IIIA | B |
| 70 | 21 | I | B |
| 74 | 21 | I | B |
| 78 | 34 | III vertex | B |
| 81 | 24 | III vertex | B |
| 2 | 37 | III vertex | C |
| 6 | 31 | III vertex | C |
| 9 | 30 | IIIA | C |
| 13 | 37 | III vertex | C |
| 17 | 25 | III vertex | C |
| 21 | 21 | II | C |
| 24 | 27 | IV | C |
| 28 | 24 | II | C |
| 32 | 30 | III vertex | C |
| 35 | 28 | III vertex | C |
| 39 | 33 | IVA | C |
| 43 | 32 | II | C |
| 50 | 38 | II | C |
| 54 | 26 | III vertex | C |
| 58 | 21 | II | C |
| 62 | 38 | II | C |
| 66 | 33 | III vertex | C |
| 68 | 20 | I | C |
| 72 | 22 | II | C |
| 76 | 24 | III vertex | C |
| 5 | 33 | IVA | Placebo |
| 12 | 32 | II | Placebo |
| 16 | 36 | III vertex | Placebo |
| 20 | 39 | II | Placebo |
| 23 | 23 | III | Placebo |
| 27 | 21 | II | Placebo |
| 31 | 35 | III vertex | Placebo |
| 34 | 23 | II | Placebo |
| 38 | 23 | III | Placebo |
| 42 | 23 | II | Placebo |
| 46 | 24 | III | Placebo |
| 49 | 22 | II | Placebo |
| 53 | 29 | III vertex | Placebo |
| 57 | 36 | IIA | Placebo |
| 61 | 38 | III vertex | Placebo |
| 65 | 21 | IIIA | Placebo |
| 71 | 20 | I | Placebo |
| 75 | 27 | III vertex | Placebo |
| 79 | 42 | IV | Placebo |
