# Supplementary material for: Molecular monitoring of short- and long-term transcriptional effects of hair growth stimulating agents
Source: PLoS One. 2024 Dec 23;19(12):e0316128. doi: 10.1371/journal.pone.0316128 (PMC11666053; doi:10.1371/journal.pone.0316128)
Supplement: S2 Table — Number of complete sample sets (corresponding to samples of the same study participant at three different time points) passing sequencing and post-processing quality control per serum and analysis (mRNA/microRNA). (DOCX) [file pone.0316128.s003.docx]

| Analysis | Serum | Sample sets |
| --- | --- | --- |
| mRNA | A | 21 |
| mRNA | B | 21 |
| mRNA | C | 20 |
| mRNA | Placebo | 19 |
| microRNA | A | 20 |
| microRNA | B | 21 |
| microRNA | C | 20 |
| microRNA | Placebo | 17 |
