# Supplementary material for: Molecular monitoring of short- and long-term transcriptional effects of hair growth stimulating agents
Source: PLoS One. 2024 Dec 23;19(12):e0316128. doi: 10.1371/journal.pone.0316128 (PMC11666053; doi:10.1371/journal.pone.0316128)
Supplement: S3 Table — Significantly differentially expressed mRNA or miRNA genes (FDR ≤ 0.05) identified in the differential expression analyses and their FDR- and fold change-values across treatment groups. The treatment groups serum B short-term, serum B long-term, serum C short-term and placebo short-term are not shown, as these showed no differentially expressed genes. Missing values indicate the gene did not meet the expression threshold in that specific treatment group and was therefore not included in the analysis. FC–Fold change. (DOCX) [file pone.0316128.s004.docx]

|  | **Serum A short-term** | | **Serum A long-term** | | **Serum C long-term** | | **Placebo long-term** | |
| --- | --- | --- | --- | --- | --- | --- | --- | --- |
| **Gene symbol** | **FDR** | **FC** | **FDR** | **FC** | **FDR** | **FC** | **FDR** | **FC** |
| *AC007098.1* | 0.290 | 1.179 | 0.031 | 1.787 |  |  |  |  |
| *ALKBH7* | 0.201 | -1.182 | 0.066 | -1.244 | 0.008 | 1.270 |  |  |
| *ANKRD13D* | 0.158 | -1.197 | 0.031 | -1.283 |  |  |  |  |
| *APOE* | 0.085 | -1.259 | 0.039 | -1.278 | 0.034 | 1.280 |  |  |
| *ATP5F1D* | 0.140 | -1.208 | 0.035 | -1.296 | 0.130 | 1.170 |  |  |
| *C1QTNF12* |  |  | 0.011 | -1.664 | 0.054 | 1.620 |  |  |
| *CALML5* | 0.076 | -1.279 | 5.30e-05 | -1.461 | 0.110 | 1.230 |  |  |
| *CDK2* | 0.140 | -1.255 | 0.015 | -1.339 |  |  | 0.538 | -1.125 |
| *CORO1B* | 0.005 | -1.328 | 0.040 | -1.246 | 0.217 | 1.130 |  |  |
| *DAPK3* | 0.003 | -1.375 | 0.001 | -1.391 | 0.034 | 1.290 |  |  |
| *DLEU2* | 0.042 | -1.359 | 0.157 | -1.254 |  |  |  |  |
| *DTX2* | 0.041 | -1.316 | 0.004 | -1.345 | 0.185 | 1.170 |  |  |
| *GNAS* |  |  | 0.046 | 1.249 |  |  |  |  |
| *GPX4* | 0.201 | -1.129 | 0.112 | -1.177 | 0.008 | 1.230 |  |  |
| *GREM1* | 0.292 | 1.230 | 0.016 | 1.794 |  |  |  |  |
| *GTPBP2* | 0.269 | 1.125 | 0.046 | 1.573 |  |  |  |  |
| *ITGA2* |  |  |  |  | 0.034 | 1.260 |  |  |
| *KAZN* | 0.268 | -1.148 | 0.046 | -1.243 |  |  |  |  |
| *LSM7* | 0.055 | -1.299 | 0.022 | -1.291 | 0.068 | 1.200 |  |  |
| *MICOS13* | 0.140 | -1.257 | 0.020 | -1.337 | 0.054 | 1.270 |  |  |
| *MPST* | 0.005 | -1.301 | 0.005 | -1.306 |  |  |  |  |
| *MRPL12* | 0.210 | -1.291 |  |  | 0.026 | 1.390 |  |  |
| *MT-TD* |  |  |  |  | 0.034 | 1.560 |  |  |
| *MVD* | 0.140 | -1.250 | 0.012 | -1.302 |  |  |  |  |
| *NCALD* | 0.293 | 1.233 | 0.046 | 1.430 |  |  |  |  |
| *NDUFB7* | 3.22e-04 | -1.364 | 2.71e-06 | -1.442 | 3.09e-06 | 1.380 |  |  |
| *NPIPB4* | 0.198 | 1.114 | 0.016 | 1.508 |  |  |  |  |
| *NUP210* | 0.048 | -1.300 |  |  |  |  |  |  |
| *OTUB1* | 0.140 | -1.198 | 0.046 | -1.228 |  |  |  |  |
| *PPP1CA* | 0.029 | -1.261 | 0.001 | -1.301 | 0.116 | 1.160 |  |  |
| *REX1BD* | 0.041 | -1.265 | 0.005 | -1.291 | 0.106 | 1.180 |  |  |
| *SGTA* | 3.83e-04 | -1.473 | 1.88e-04 | -1.428 | 0.080 | 1.260 |  |  |
| *STK11* | 0.042 | -1.307 | 0.182 | -1.192 | 0.109 | 1.220 |  |  |
| *TDRD3* |  |  | 0.027 | 1.269 | 0.282 | 1.130 |  |  |
| *TMEM217* | 0.129 | -1.419 | 0.046 | -1.483 |  |  | 0.433 | -1.228 |
| *TMEM250* | 0.030 | -1.267 | 0.005 | -1.270 |  |  |  |  |
| *TSPAN4* | 0.301 | -1.319 | 0.046 | -1.632 | 0.067 | 1.530 | 0.489 | 1.303 |
| *UBOX5* |  |  |  |  | 0.034 | 1.540 |  |  |
| *WDR34* | 0.103 | -1.325 |  |  | 0.026 | 1.260 |  |  |
| *ZBTB20* |  |  |  |  | 0.008 | 1.270 | 0.336 | 1.163 |
| *ZNF141* |  |  |  |  | 0.026 | 1.360 |  |  |
| *ZNF302* | 0.223 | 1.221 | 0.014 | 1.606 |  |  |  |  |
| *hsa-miR-128-3p* |  |  |  |  | 0.026 | -1.429 |  |  |
| *hsa-miR-152-3p* |  |  |  |  | 0.006 | 1.212 | 0.292 | 1.139 |
| *hsa-miR-197-3p* | 0.810 | -1.151 |  |  | 0.006 | -1.311 |  |  |
| *hsa-miR-221-3p* |  |  |  |  | 0.006 | 1.242 |  |  |
| *hsa-miR-3168* |  |  |  |  | 0.038 | -1.285 |  |  |
| *hsa-miR-423-3p* |  |  |  |  | 0.036 | -1.201 |  |  |
| *hsa-miR-452-5p* |  |  |  |  | 4.69e-04 | 1.320 |  |  |
| *hsa-miR-509-3-5p* |  |  |  |  | 0.049 | 1.236 |  |  |
| *hsa-miR-92a-1-5p* |  |  |  |  | 0.023 | -1.328 |  |  |
| *hsa-miR-9985* |  |  | 0.487 | 1.325 | 0.078 | 1.445 | 0.025 | 1.575 |
| *hsa-miR-99a-5p* |  |  |  |  | 0.032 | 1.218 |  |  |
