# Supplementary material for: Molecular monitoring of short- and long-term transcriptional effects of hair growth stimulating agents
Source: PLoS One. 2024 Dec 23;19(12):e0316128. doi: 10.1371/journal.pone.0316128 (PMC11666053; doi:10.1371/journal.pone.0316128)
Supplement: S4 Table — Significant associations (FDR ≤ 0.05) identified between pathway-based polygenic risk scores (pPRS) and serum-mediated effects on gene expression of DE mRNA or miRNA genes (P ≤ 0.05 in any of serum A, B or C). (DOCX) [file pone.0316128.s005.docx]

| **pPRS** | **Gene** | **beta** | **P** | **FDR** | **Serum** |
| --- | --- | --- | --- | --- | --- |
| FOXA2 pathway | AC026464.6 | -391.5 | 1.6×10^−10^ | 2.0×10^−4^ | A (short-term) |
| S1P receptor signal transduction | WNT5A | 203.0 | 2.4×10^−8^ | 1.5×10^−2^ | A (short-term) |
| Heme biosynthesis | AL353691.2 | 557.2 | 4.0×10^−14^ | 5.2×10^−8^ | A (long-term) |
| Cancer immunotherapy by CTLA4 blockade | AF228730.5 | 512.1 | 1.2×10^−8^ | 4.9×10^−3^ | B (long-term) |
| Microglia pathogen phagocytosis pathway | AF228730.5 | 512.1 | 1.2×10^−8^ | 4.9×10^−3^ | B (long-term) |
