# Supplementary figures and images for: Molecular monitoring of short- and long-term transcriptional effects of hair growth stimulating agents
Source: PLoS One. 2024 Dec 23;19(12):e0316128. doi: 10.1371/journal.pone.0316128 (PMC11666053; doi:10.1371/journal.pone.0316128)

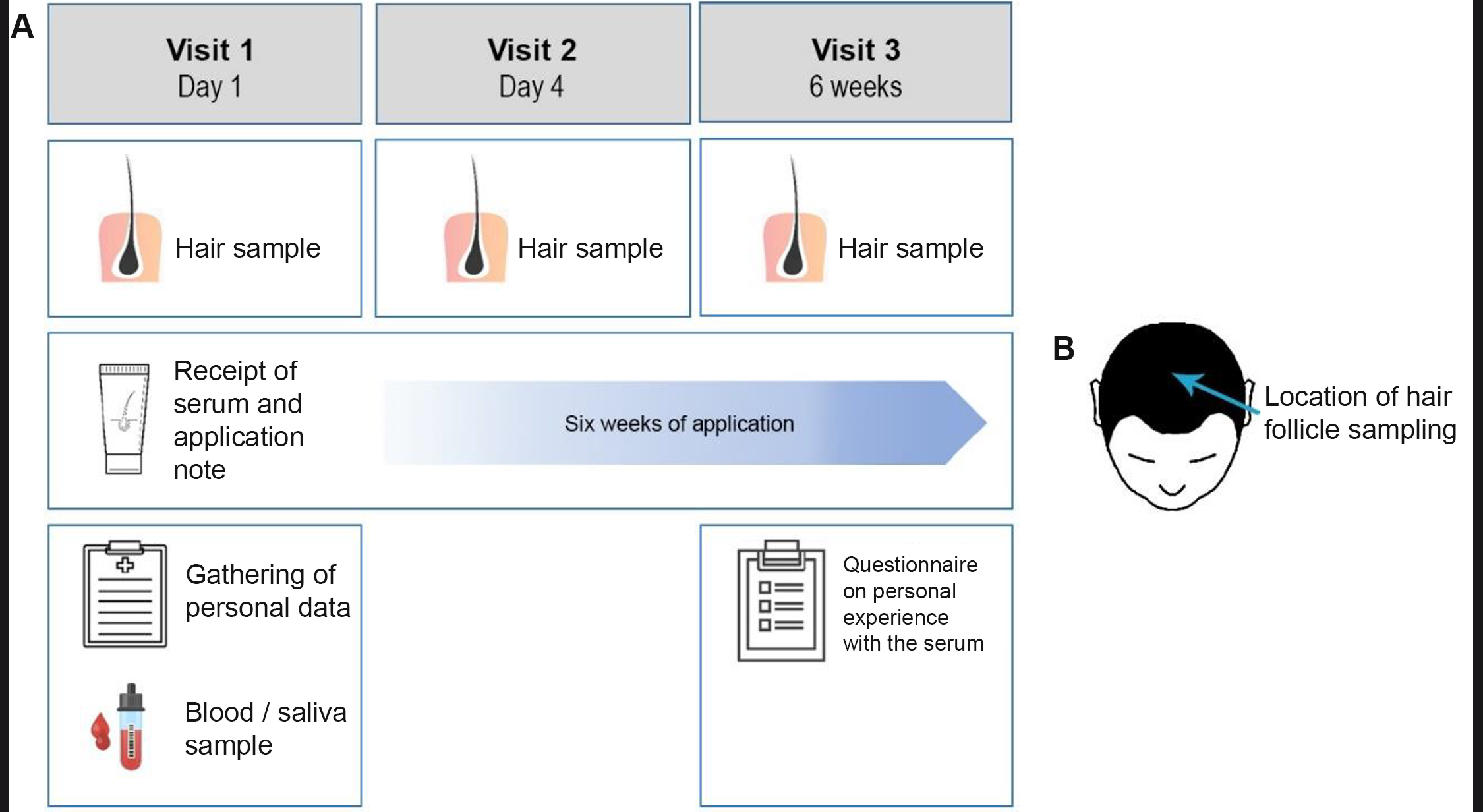

Supplement: S1 Fig — (A) Hair follicles were extracted from participants at three visits. On the first visit, a blood sample was drawn, personal information was recorded and participants were given a serum or placebo to be used for the following six weeks. On the third visit, participants answered a questionnaire on their experience with the serum. (B) Schematic image of the location from which hair follicles were sampled, as denoted by the blue arrow. Approximately 50 hair follicles were plucked from the center of the scalp. (TIFF) [file pone.0316128.s006.tiff]

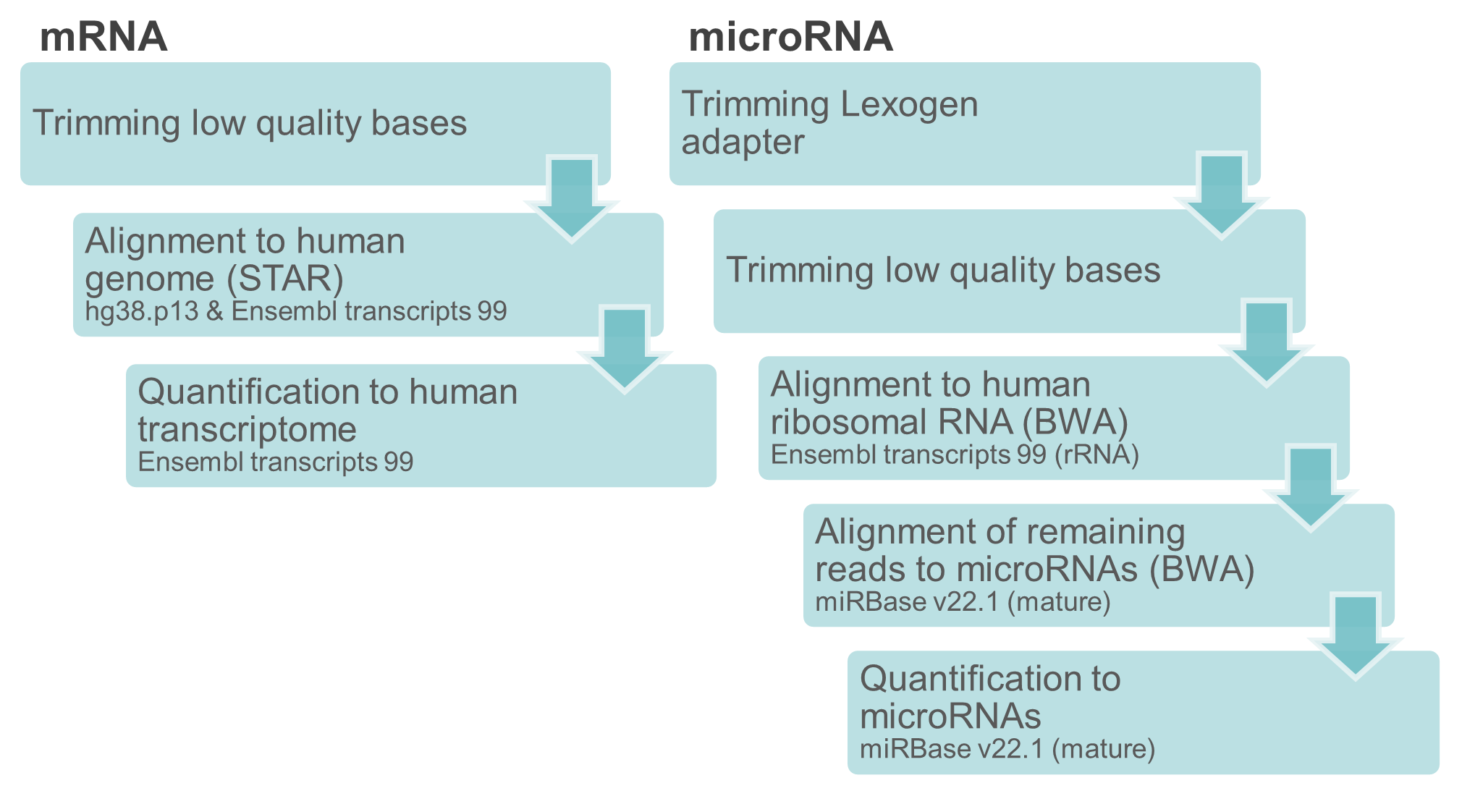

Supplement: S2 Fig — (TIFF) [file pone.0316128.s007.tiff]

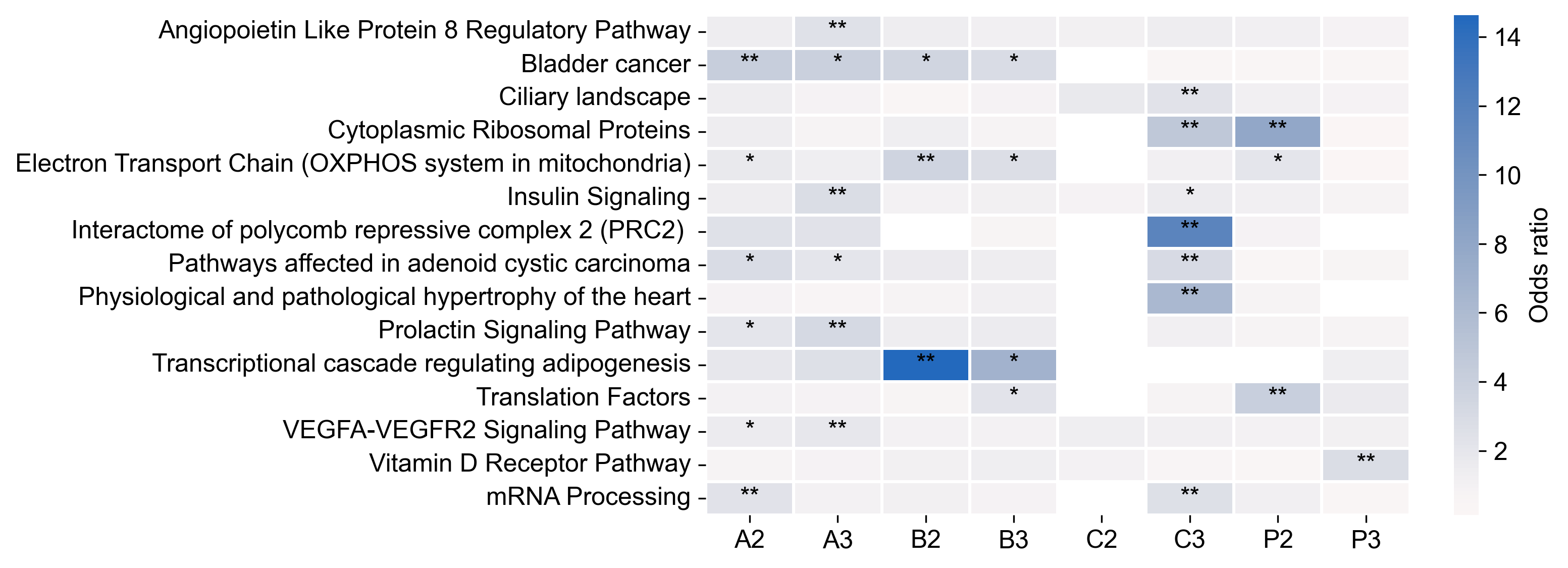

Supplement: S3 Fig — Only pathways which are significantly enriched (FDR ≤ 0.05) in at least one treatment group are shown. Treatment groups refer to serums A, B, C and placebo (P), at time points 2 (short-term treatment) and 3 (long-term treatment). ** indicates significant enrichment (FDR ≤ 0.05), * indicates nominally significant enrichment (p ≤ 0.05). The enrichment odds ratio is shown on a color scale. (TIFF) [file pone.0316128.s008.tiff]

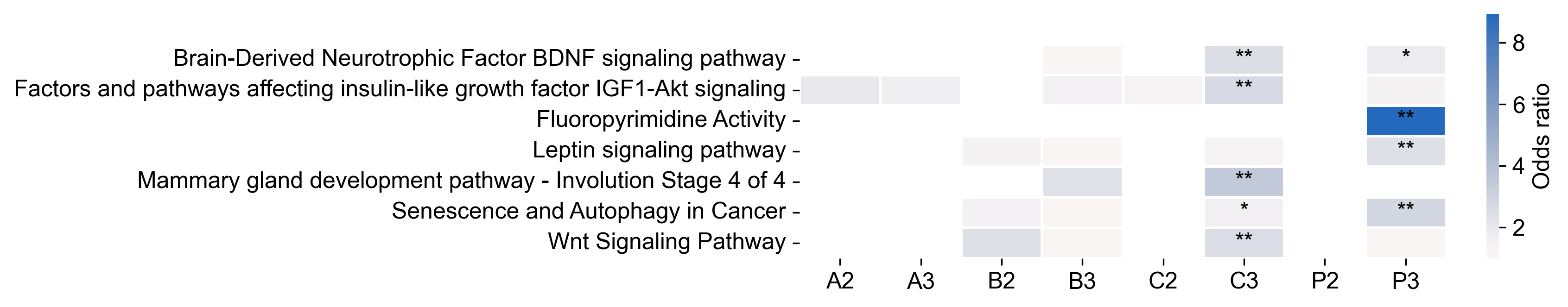

Supplement: S4 Fig — Only pathways which are significantly enriched (FDR ≤ 0.05) in at least one treatment group are shown. Treatment groups refer to serums A, B, C and placebo (P), at time points 2 (short-term treatment) and 3 (long-term treatment). ** indicates significant enrichment (FDR ≤ 0.05), * indicates nominally significant enrichment (p ≤ 0.05). The enrichment odds ratio is shown on a color scale. (TIFF) [file pone.0316128.s009.tiff]
